# Molecular cloning using polymerase chain reaction, an educational guide for cellular engineering

**DOI:** 10.1186/1754-1611-9-2

**Published:** 2015-01-19

**Authors:** Sayed Shahabuddin Hoseini, Martin G Sauer

**Affiliations:** Departments of Pediatric Hematology, Oncology and Blood Stem Cell Transplantation, Hannover, Germany; Hannover Center for Transplantation Research, Hannover, Germany; Department of Pediatric Hematology and Oncology, Medizinische Hochschule Hannover, OE 6780, Carl-Neuberg-Strasse 1, 30625 Hannover, Germany

**Keywords:** Cloning, Polymerase chain reaction (PCR), Recombinant DNA, Biological engineering, Educational guide, Transduction, Transfection

## Abstract

**Background:**

Over the last decades, molecular cloning has transformed biological sciences. Having profoundly impacted various areas such as basic science, clinical, pharmaceutical, and environmental fields, the use of recombinant DNA has successfully started to enter the field of cellular engineering. Here, the polymerase chain reaction (PCR) represents one of the most essential tools. Due to the emergence of novel and efficient PCR reagents, cloning kits, and software, there is a need for a concise and comprehensive protocol that explains all steps of PCR cloning starting from the primer design, performing PCR, sequencing PCR products, analysis of the sequencing data, and finally the assessment of gene expression. It is the aim of this methodology paper to provide a comprehensive protocol with a viable example for applying PCR in gene cloning.

**Results:**

Exemplarily the sequence of the tdTomato fluorescent gene was amplified with PCR primers wherein proper restriction enzyme sites were embedded. Practical criteria for the selection of restriction enzymes and the design of PCR primers are explained. Efficient cloning of PCR products into a plasmid for sequencing and free web-based software for the consecutive analysis of sequencing data is introduced. Finally, confirmation of successful cloning is explained using a fluorescent gene of interest and murine target cells.

**Conclusions:**

Using a practical example, comprehensive PCR-based protocol with important tips was introduced. This methodology paper can serve as a roadmap for researchers who want to quickly exploit the power of PCR-cloning but have their main focus on functional *in vitro* and *in vivo* aspects of cellular engineering.

**Electronic supplementary material:**

The online version of this article (doi:10.1186/1754-1611-9-2) contains supplementary material, which is available to authorized users.

## Background

Various techniques were introduced for assembling new DNA sequences [1–3], yet the use of restriction endonuclease enzymes is the most widely used technique in molecular cloning. Whenever compatible restriction enzyme sites are available on both, insert and vector DNA sequences, cloning is straightforward; however, if restriction sites are incompatible or if there is even no restriction site available in the vicinity of the insert cassette, cloning might become more complex. The use of PCR primers, in which compatible restriction enzyme sites are embedded, can effectively solve this problem and facilitate multistep cloning procedures.

Although PCR cloning has been vastly used in biological engineering [4–8], practical guides explaining all necessary steps and tips in a consecutive order are scarce. Furthermore, the emergence of new high-fidelity DNA polymerases, kits, and powerful software makes the process of PCR cloning extremely fast and efficient. Here we sequentially explain PCR cloning from the analysis of the respective gene sequence, the design of PCR primers, performing the PCR procedure itself, sequencing the resulting PCR products, analysis of sequencing data, and finally the cloning of the PCR product into the final vector.

## Results and discussion

### Choosing proper restriction enzymes based on defined criteria

In order to proceed with a concise example, tdTomato fluorescent protein was cloned into an alpharetroviral vector. Consecutively, a murine leukemia cell line expressing tdTomato was generated. This cell line will be used to track tumor cells upon injection into mice in preclinical immunotherapy studies. However, this cloning method is applicable to any other gene. To begin the cloning project, the gene of interest (GOI) should be analyzed. First, we check whether our annotated sequence has a start codon (ATG, the most common start codon) and one of the three stop codons (TAA, TAG, TGA). In case the gene was previously manipulated or fused to another gene (e.g. via a 2A sequence), it happens that a gene of interest might not have a stop codon [9]. In such cases, a stop codon needs to be added to the end of your annotated sequence. It is also beneficial to investigate whether your GOI contains an open reading frame (ORF). This is important since frequent manipulation of sequences either by software or via cloning might erroneously add or delete nucleotides. We use Clone Manager software (SciEd) to find ORFs in our plasmid sequences; however, there are several free websites you can use to find ORFs including the NCBI open reading frame finder ( http://www.ncbi.nlm.nih.gov/gorf/gorf.html).

**Figure 1:**
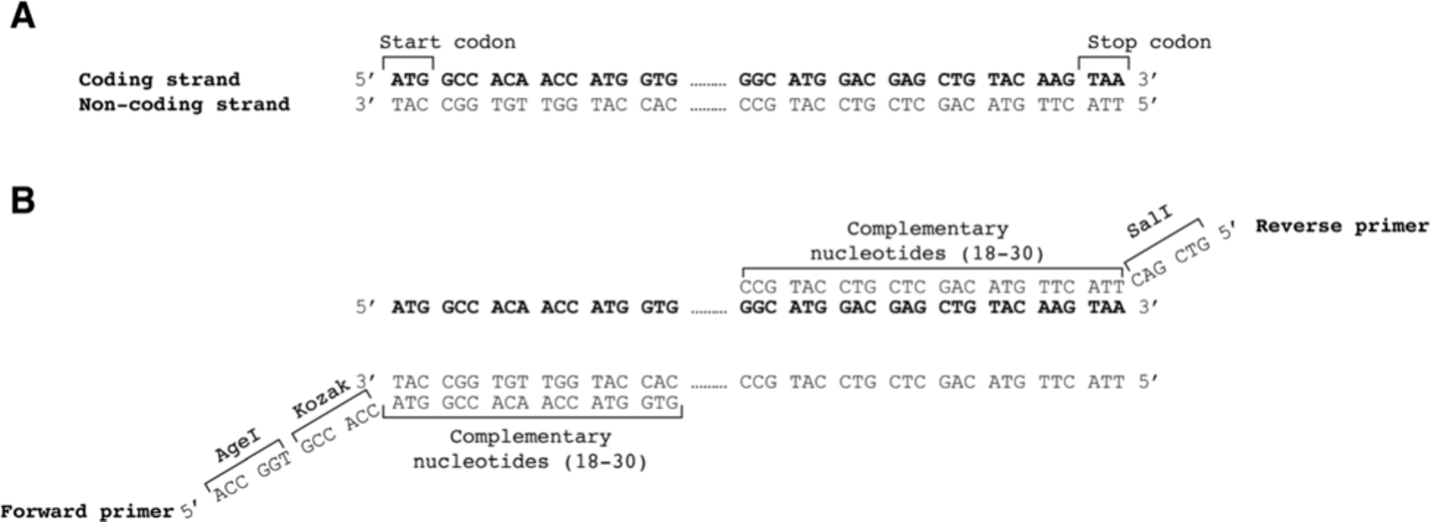
**Overview of the start and the end of the gene of interest. (A)** The nucleotide sequences at the start and the end of the tdTomato gene are shown. The coding strand nucleotides are specified in bold **(B)** The nucleotide sequences of the forward and reverse primers containing proper restriction enzyme sites and the Kozak sequence are shown.

The tdTomato gene contains ATG start codon and TAA stop codon (Figure 1). The size of the tdTomato gene is 716 bp.

**Figure 2:**
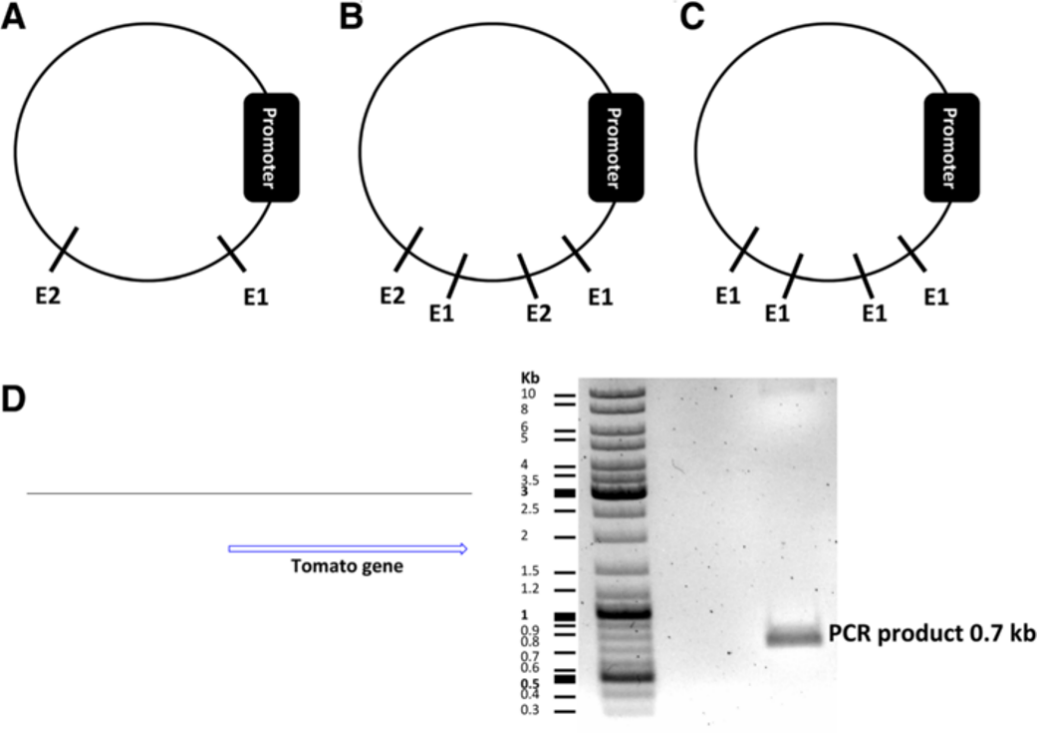
**Choosing proper restriction enzymes based on defined criteria for PCR cloning. (A)** Two single-cutter restriction enzymes (E1 and E2) are located downstream of the promoter. **(B)** E1 and E2 restriction enzymes cut the plasmid downstream of the promoter several (here two times for each enzyme) times. **(C)** The E1 restriction enzyme cuts the plasmid downstream of the promoter more than once. **(D)** The PCR product, which contains the tdTomato gene and the restriction enzyme sites, was run on a gel before being extracted for downstream applications.

In a next step, PCR primers that include proper restriction enzyme sites need to be designed for the amplification of the GOI. Several criteria should be considered in order to choose the optimal restriction enzymes. First, binding sites for restriction enzymes should be ideally available at a multiple cloning site within the vector. Alternatively they can be located downstream of the promoter in your vector sequence. Restriction enzymes should be single cutters (single cutters target one restriction site only within a DNA sequence) (Figure 2A). If they are double or multiple cutters, they should cut within a sequence that is not necessary for proper functioning of the vector plasmid and will finally be removed (Figure 2B). It is also possible to choose one double cutter or multiple cutter enzymes cutting the vector downstream of the promoter and also not within a vital sequence of the plasmid (Figure 2C). Double cutter or multiple cutter enzymes have two or more restriction sites on a DNA sequence, respectively. Cutting the vector with double or multiple cutters would give rise to two identical ends. In such a case, the insert cassette should also contain the same restriction enzyme sites on both of its ends. Therefore, when the insert and vector fragments are mixed in a ligation experiment, the insert can fuse to the vector in either the right orientation (from start codon to stop codon) or reversely (from stop codon to start codon). A third scenario can occur, if the vector fragment forms a self-ligating circle omitting the insert at all. Once the DNA has been incubated with restriction enzymes, dephosphorylation of the 5′ and 3′ ends of the vector plasmid using an alkaline phosphatase enzyme will greatly reduce the risk of self-ligation [10]. It is therefore important to screen a cloning product for those three products (right orientation, reverse orientation, self-ligation) after fragment ligation.

Second, due to higher cloning efficiency using sticky-end DNA fragments, it is desirable that at least one (better both) of the restriction enzymes is a so-called sticky-end cutter. Sticky end cutters cleave DNA asymmetrically generating complementary cohesive ends. In contrast, blunt end cutters cut the sequence symmetrically leaving no overhangs. Cloning blunt-end fragments is more difficult. Nevertheless, choosing a higher insert/vector molar ratio (5 or more) and the use 10% polyethylene glycol (PEG) can improve ligation of blunt-end fragments [11].

Third, some restriction enzymes do not cut methylated DNA. Most of the strains of *E. coli* contain Dam or Dcm methylases that methylate DNA sequences. This makes them resistant to methylation-sensitive restriction enzymes [12]. Since vector DNA is mostly prepared in *E. coli*, it will be methylated. Therefore avoiding methylation-sensitive restriction enzymes is desirable; however, sometimes the isoschizomer of a methylation-sensitive restriction enzyme is resistant to methylation. For example, the *Acc*65I enzyme is sensitive while its isoschizomer *kpn*I is resistant to methylation [13]. Isoschizomers are restriction enzymes that recognize the same nucleotide sequences. If there remains no other option than using methylation-sensitive restriction enzymes, the vector DNA needs to be prepared in *dam*^*−*^*dcm*^*−*^*E. coli* strains. A list of these strains and also common *E. coli* host strains for molecular cloning is summarized in Table 1. Information regarding the methylation sensitivity of restriction enzymes is usually provided by the manufacturer.

**Table 1:** **Common** ***E. coli*** **host strains in gene cloning**

Application	***E. coli***strain
Transformation of large plasmids	GeneHogs, XL10 Gold, STBL4, SURE
Generation of single-stranded (ssDNA)	INV110, JM109, JS5, NM522, SCS110, SURE, STBL4, XL10 Gold, XL1 Blue, TG1
For storage of plasmids that tend to recombine	DH10B, DH5α, STBL2, STBL3, STBL4, SURE, GeneHogs, Hb101, JM109, JS5, XL10 Gold, XL1 Blue, GC5, GC10
Rapid cloning (fast cell growth)	Mach1
Cloning of unstable plasmids	SURE, STBL2, STBL3, STBL4
High efficiency cloning for library construction	XL10-Gold, MegaX, DH10B
Blue/white screening	DH10B, DH5α, MC1061, TOP10, XL1 Blue, Hb101, NM522, SCS110, STBL4, SURE, XL10 Gold, GeneHogs, INV110, JM109, JS5, GC5, GC10
For site-directed mutagenesis	XL-*mutS*, BMH 71–18 *mutS*, ES1301 *mutS*
For random mutagenesis	XL1-Red
For expression of toxic genes	ABLE C, ABLE K
General cloning and storage of plasmids	DH10B, DH5α, MC1061, TOP10, XL1-Blue, Hb101
For proliferation of plasmids encoding the ccdB toxic gene (important in Gateway cloning)	ccdB survival, DB3.1, XL1 Blue, JM109, DH5α
For generation of unmethylated DNA to be cut with methylation-sensitive restriction enzymes	JM110, ER2925, INV110, DM1, SCS110, E4109S

Data are derived from enzyme providers’ data sheets, the following website, and this reference [14]. http://openwetware.org/wiki/E._coli_genotypes.

Fourth, it makes cloning easier if the buffer necessary for the full functionality of restriction enzymes is the same because one can perform double restriction digest. This saves time and reduces the DNA loss during purification. It may happen that one of the restriction enzymes is active in one buffer and the second enzyme is active in twice the concentration of the same buffer. For example the *Nhe*I enzyme from Thermo Scientific is active in Tango 1X buffer (Thermo Scientific) and *Eco*R1 enzyme is active in Tango 2X buffer (Thermo Scientific). In such cases, the plasmid DNA needs to be first digested by the enzyme requiring the higher buffer concentration (here *Eco*R1). This will be followed by diluting the buffer for the next enzyme (requiring a lower concentration (here *Nhe*I)) in the same buffer. However, the emergence of universal buffers has simplified the double digest of DNA sequences [15]. In our example the vector contains the *Age*I and *Sal*I restriction sites. These enzyme sites were used for designing PCR primers (Figure 1). It is essential for proper restriction enzyme digestion that the plasmid purity is high. DNA absorbance as measured by a spectrophotometer can be used to determine the purity after purification. DNA, proteins, and solvents absorb at 260 nm, 280 nm, and 230 nm, respectively. An OD 260/280 ratio of >1.8 and an OD 260/230 ratio of 2 to 2.2 is considered to be pure for DNA samples [16]. The OD 260/280 and 260/230 ratios of our exemplary plasmid preparations were 1.89 and 2.22, respectively. We observed that the purity of the gel-extracted vector and insert DNA fragments were lower after restriction digest; ligation works even in such cases, however, better results can be expected using high-purity fragments.

The following plasmid repository website can be useful for the selection of different vectors (viral expression and packaging, empty backbones, fluorescent proteins, inducible vectors, epitope tags, fusion proteins, reporter genes, species-specific expression systems, selection markers, promoters, shRNA expression and genome engineering): http://www.addgene.org/browse/.

A collection of cloning vectors of *E. coli* is available under the following website: http://www.shigen.nig.ac.jp/ecoli/strain/cvector/cvectorExplanation.jsp.

### Designing cloning primers based on defined criteria

**Figure 3:**
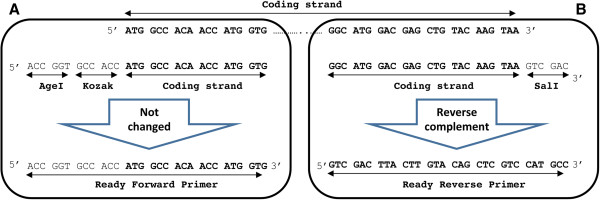
**Designing primers based on defined criteria for PCR cloning. (A-B)** Sequences of the forward and the reverse primer are depicted. The end of the coding strand is to be converted into the reverse complement format for the reverse primer design. For more information, please see the text.

For PCR primer design, check the start and stop codons of your GOI. Find the sequence of the desired restriction enzymes (available on the manufacturers’ websites) for the forward primer (Figure 3A). It needs to be located before the GOI (Figure 1B). The so-called Kozak sequence is found in eukaryotic mRNAs and improves the initiation of translation [17]. It is beneficial to add the Kozak sequence (GCCACC) before the ATG start codon since it increases translation and expression of the protein of interest in eukaryotes [18]. Therefore, we inserted GCCACC immediately after the restriction enzyme sequence *Age*I and before the ATG start codon. Then, the first 18 to 30 nucleotides of the GOI starting from the ATG start codon are added to the forward primer sequence. These overlapping nucleotides binding to the template DNA determine the annealing temperature (Tm). The latter is usually higher than 60°C. Here, we use Phusion high-fidelity DNA polymerase (Thermo Scientific). You can use the following websites for determination of the optimal Tm: http://www.thermoscientificbio.com/webtools/tmc/.

https://www.neb.com/tools-and-resources/interactive-tools/tm-calculator.

The Tm of our forward primer is 66°C.

Choose the last 18 to 30 nucleotides including the stop codon of your GOI for designing the reverse primer (Figure 3B). Then calculate the Tm for this sequence which should be above 60°C and close to the Tm of the forward primer. Tm of the overlapping sequence of our reverse primer was 68°C. Then, add the target sequence of the second restriction enzyme site (in this case *Sal*I) immediately after the stop codon. Finally, convert this assembled sequence to a reverse-complement sequence. The following websites can be used to determine the sequence of the reverse primer:

http://reverse-complement.com/

http://www.bioinformatics.org/sms/rev_comp.htmlThis is important since the reverse primer binds the coding strand and therefore its sequence (5′ → 3′) must be reverse-complementary to the sequence of the coding strand (Figure 1A).

### Performing PCR using proofreading polymerases

Since the PCR reaction follows logarithmic amplification of the target sequence, any replication error during this process will be amplified. The error rate of non-proofreading DNA polymerases, such as the Taq polymerase, is about 8 × 10^−6^ errors/bp/PCR cycle [19]; however, proofreading enzymes such as Phusion polymerase have a reported error rate of 4.4 × 10^−7^ errors/bp/PCR cycle. Due to its superior fidelity and processivity [20–22], the Phusion DNA polymerase was used in this example. It should be noted that Phusion has different temperature requirements than other DNA polymerases. The primer Tm for Phusion is calculated based on the Breslauer method [23] and is higher than the Tm using Taq or pfu polymerases. To have optimal results, the Tm should be calculated based on information found on the website of the enzyme providers. Furthermore, due to the higher speed of Phusion, 15 to 30 seconds are usually enough for the amplification of each kb of the sequence of interest.

After the PCR, the product needs to be loaded on a gel (Figure 2D). The corresponding band needs to be cut and the DNA extracted. It is essential to sequence the PCR product since the PCR product might include mutations. There are several PCR cloning kits available some of which are shown in Table 2. We used the pJET1.2/blunt cloning vector (Thermo Scientific, patent publication: US 2009/0042249 A1, Genbank accession number EF694056.1) and cloned the PCR product into the linearized vector. This vector contains a lethal gene (*eco47IR*) that is activated in case the vector becomes circularized. However, if the PCR product is cloned into the cloning site within the lethal gene, the latter is disrupted allowing bacteria to grow colonies upon transformation. Circularized vectors not containing the PCR product express the toxic gene, which therefore kills bacteria precluding the formation of colonies. Bacterial clones are then to be cultured, plasmid DNA consecutively isolated and sequenced. The quality of isolated plasmid is essential for optimal sequencing results. We isolated the plasmid DNA from a total of 1.5 ml cultured bacteria (yield 6 μg DNA; OD 260/280 = 1.86; OD 260/230 = 2.17) using a plasmid mini-preparation kit (QIAGEN). The whole process of PCR, including cloning of the PCR product into the sequencing vector and transfection of bacteria with the sequencing vector can be done in one day. The next day, bacterial clones will be cultured overnight before being sent for sequencing.

**Table 2:** **Common vectors in gene cloning**

Plasmid name	Advantages	Disadvantages	References
pBR322	Small size (4.4 kb), variety of cloning sites, medium copy number (15–20)		[24, 25]
pUC18 and pUC19	Small size (2.7 kb), high copy number (500–700), multiple cloning site, sequencing using M13 primers	Not good if target protein is toxic or for membrane proteins, needs blue/white screen	[24, 26]
pLG338	General purpose plasmid vector, size 7.3 kb, low copy number (6–8), genes coding for membrane and regulatory proteins which cannot be cloned into high-copy-number plasmids		[27]
pMiniT	Inserted PCR product disrupts a toxic minigene, no blue/white selection required, works for both blunt end and single-base overhang-containing PCR products		NEB
pCR™4Blunt-TOPO®	Inserted PCR product disrupts *ccd*B toxic gene [28], works based on topoisomerase I [29], no blue/white selection required	Needs blunt-end PCR products	Life Technologies
pDrive	Size 3.8 kb	Needs single A overhangs, not usable for proofreading DNA polymerases, needs blue/white screening	QIAGEN
StrataClone Blunt PCR Cloning Vector pSC-B-amp/kan	Uses the DNA topoisomerase I and the DNA recombination activity of Cre recombinase, up to 9 kb PCR product size	Needs blunt-end PCR products, needs special competent cells for transformation (cells expressing Cre recombinase)	Agilent Technologies
pJET1.2/blunt	Inserted PCR product disrupts the toxic gene *eco47IR*, no need for blue/white screen, works for both blunt end and single-base overhang-containing PCR products, up to 10 kb PCR product size		Thermo Scientific

Data are derived from vector providers or the cited references. pBR322, pUC18, pUC19, and pLG338 are cloning vectors and the rest are PCR sequencing vectors.

### Analysis of sequencing data

Sequencing companies normally report sequencing data as a FASTA file and also as ready nucleotide sequences via email. For sequence analysis, the following websites can be used:

http://blast.ncbi.nlm.nih.gov/Blast.cgi

http://xylian.igh.cnrs.fr/bin/align-guess.cgi

**Figure 4:**
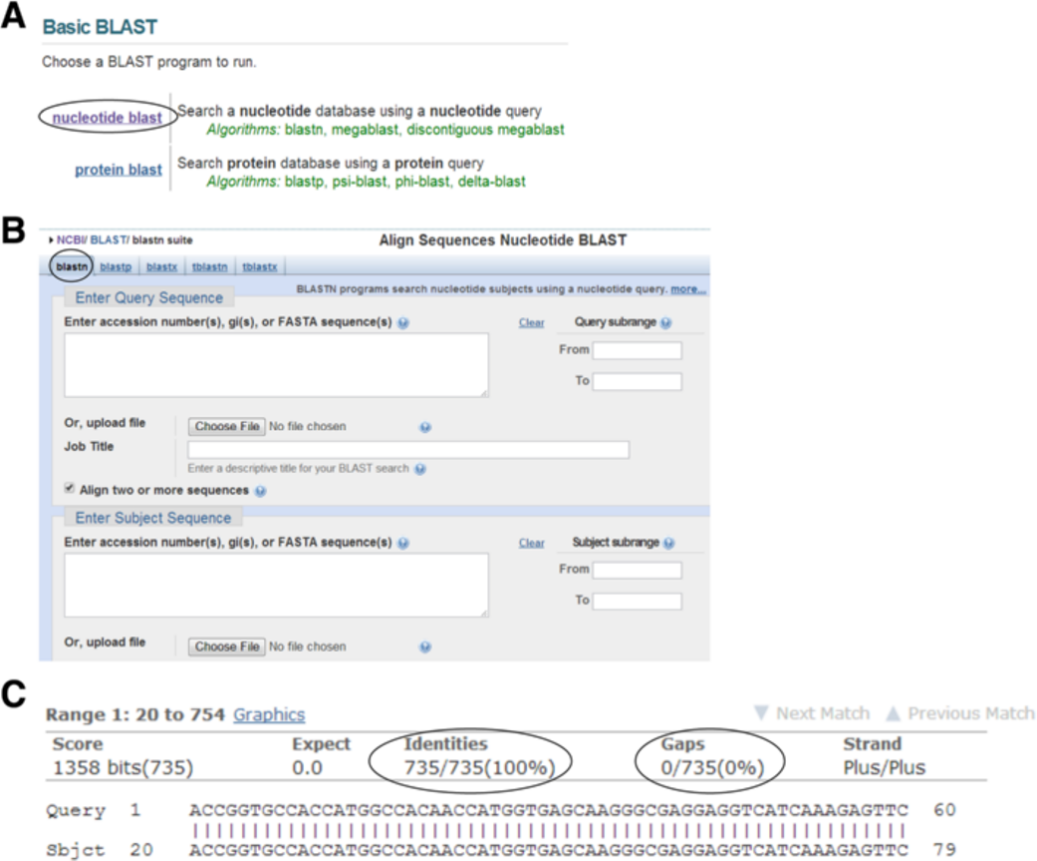
**Sequence analysis of the PCR product using the NCBI BLAST platform. (A)** On the NCBI BLAST webpage, the “nucleotide blast” option is chosen (marked by the oval line). **(B)** The “blastn” option appears by default (marked by the circle). The sequence of the gene of interest (flanked by the restriction sites as previously designed for the PCR primers) and the PCR product are to be inserted to the “Enter Query Sequence” and “Enter Subject Sequence” boxes. Sequences can also be uploaded as FASTA files. **(C)** Nucleotide alignment of the first 60 nucleotides is shown. Two important items for sequence analysis are marked by oval lines.

Here we will focus on the first website. On this website page, click on the “nucleotide blast” option (Figure 4A). A new window opens. By default, the “blastn” (blast nucleotide sequences) option is marked (Figure 4B). Then check the box behind “Align two or more sequences”. Now two boxes will appear. In the “Enter Query Sequence” box (the upper box), insert the desired sequence of your gene of interest, which is flanked by the restriction sites you have already designed for your PCR primers. In the “Enter Subject Sequence” box (the lower box), enter the sequence or upload the FASTA file you have received from the sequencing company. Then click the “BLAST” button at the bottom of the page. After a couple of seconds, the results will be shown on another page. A part of the alignment data is shown in Figure 4C. For interpretation, the following points should be considered: 1) the number of identical nucleotides (shown under the “Identities” item) must be equal to the nucleotide number of your gene of interest. In our example, the number of nucleotides of the tdTomato gene together with those of the restriction enzyme sites and the Kozak sequence was 735. This equals the reported number (Figure 4C). 2) The sequence identity (under the “Identities” item) should be 100%. Occasionally, the sequence identity is 100% but the number of identical nucleotides is lower than expected. This can happen if one or more of the initial nucleotides are absent. Remember, all sequencing technologies have an error rate. For Sanger sequencing, this error rate is reported to range from 0.001% to 1% [30–33]. Nucleotide substitution, deletion or insertion can be identified by analyzing the sequencing results [34]. Therefore, if the sequence identity does not reach 100%, the plasmid should be resequenced in order to differentiate errors of the PCR from simple sequencing errors. 3) Gaps (under the “Gaps” item) should not be present. If gaps occur, the plasmid should be resequenced.

The average length of a read, or read length, is at least 800 to 900 nucleotides for Sanger sequencing [35]. For the pJET vector one forward and one reverse primer need to be used for sequencing the complete gene. These primers can normally cover a gene size ranging up to 1800 bp. If the size of a gene is larger than 1800, an extra primer should be designed for each 800 extra nucleotides. Since reliable base calling does not start immediately after the primer, but about 45 to 55 nucleotides downstream of the primer [36], the next forward primer should be designed to start after about 700 nucleotides from the beginning of the gene. Different websites, including the following, can be used to design these primers:

http://www.ncbi.nlm.nih.gov/tools/primer-blast/

http://www.yeastgenome.org/cgi-bin/web-primer

http://www.genscript.com/cgi-bin/tools/sequencing_primer_design

Being 735 bp in length, the size of the PCR product in this example was well within the range of the pJET sequencing primers.

**Figure 5:**
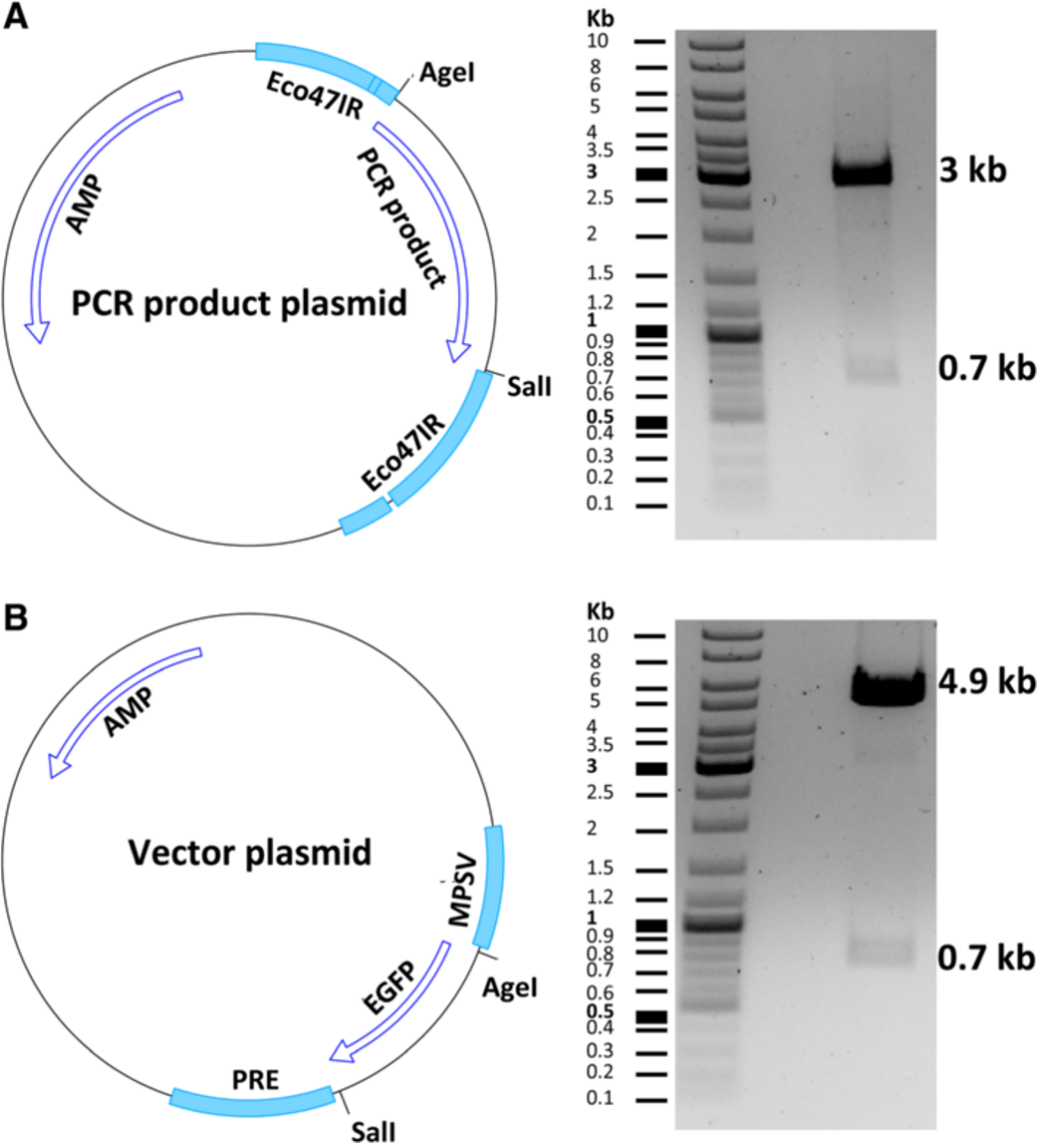
**Vector and insert plasmid maps A)** Illustration of the CloneJET plasmid containing the PCR product. Insertion of the PCR product in the cloning site of the plasmid disrupts the integrity of the toxic gene *eco47IR* and allows the growth of transgene positive clones. The plasmid was cut with the *Age*I and *Sal*I enzymes generating two fragments of 3 kb and 0.7 kb in size. The 0.7 kb fragment (tdTomato gene) was used as the insert for cloning. **(B)** Illustration of the vector plasmid. The plasmid was cut with the *Age*I and *Sal*I enzymes generating two fragments of 4.9 kb and 0.7 kb in size. The 4.9 kb fragment was used as the vector for cloning. AMP: Ampicillin resistance gene; PRE: posttranscriptional regulatory element; MPSV: myeloproliferative sarcoma virus promoter.

**Figure 6:**
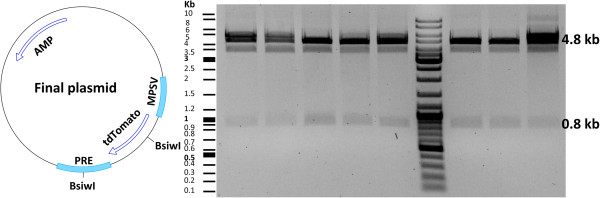
**Screening of the final plasmid with restriction enzymes.** Illustration of the final plasmid is shown. For screening, the plasmid was cut with the *Bsiw*I enzyme generating two fragments of 4.8 kb and 0.8 kb in size. AMP: Ampicillin resistance gene; PRE: posttranscriptional regulatory element; MPSV: myeloproliferative sarcoma virus promoter.

After choosing the sequence-verified clone, vector and insert plasmids were digested by the *Age*I and *Sal*I restriction enzymes (Figure 5). This was followed by gel purification and ligation of the fragments. Transformation of competent *E. coli* with the ligation mixture yielded several clones that were screened by restriction enzymes. We assessed eight clones, all of which contained the tdTomato insert (Figure 6). It is important to pick clones that are large. Satellite clones might not have the right construct. We used a fast plasmid mini-preparation kit (Zymo Research) to extract the plasmid from 0.6 ml bacterial suspension. The yield and purity were satisfying for restriction enzyme-based screening (2.3 μg DNA; OD 260/280 = 1.82; OD 260/230 = 1.41). For large-scale plasmid purification, a maxi-preparation kit (QIAGEN) was used to extract the plasmid from 450 ml of bacterial culture (yield 787 μg DNA; OD 260/280 = 1.89; OD 260/230 = 2.22). The expected yield of a pBR322-derived plasmid isolation from 1.5 ml and 500 ml bacterial culture is about 2-5 μg and 500-4000 μg of DNA, respectively [37].

Some plasmids tend to recombine inside the bacterial host creating insertions, deletions and recombinations [38]. In these cases, using a recA-deficient *E. coli* can be useful (Table 1). Furthermore, if the GOI is toxic, incubation of bacteria at lower temperatures (25-30°C) and using ABLE C or ABLE K strains might circumvent the problem.

### Viral production and transduction of target cells

**Figure 7:**
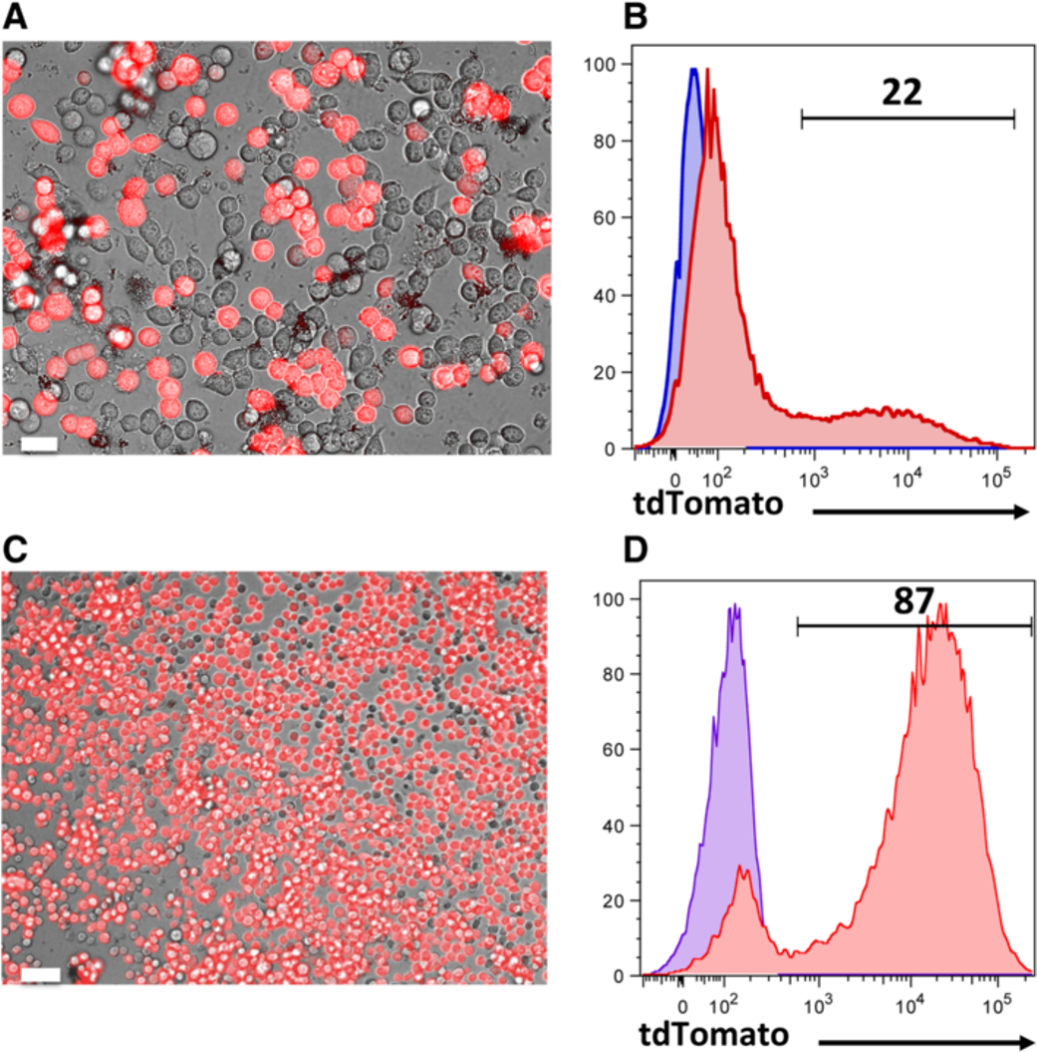
**Assessing** ***in vitro*** **expression of the cloned gene. (A, B)** HEK293T cells were transfected with Gag/Pol, VSVG, and tdTomato plasmids. The expression of the tdTomato gene was assessed using a fluorescence microscope. Fluorescent images were superimposed on a bright-field image for the differentiation of positively transduced cells. Transfection efficiency was determined based on the expression of tdTomato after 24 hours. Non-transfected HEK293T cells were used as controls (blue histogram). **(C, D)** The murine leukemia cell line C1498 was transduced with fresh virus. Four days later, transgene expression was assessed by fluorescence microscopy **(C)** and flow cytometry **(D)**. Non-transduced C1498 cells were used as controls (blue histogram). Scale bars represent 30 μm.

To investigate the *in vitro* expression of the cloned gene, HEK293T cells were transfected with plasmids encoding the tdTomato gene, alpharetroviral Gag/Pol, and the vesicular stomatitis virus glycoprotein (VSVG) envelope. These cells, which are derived from human embryonic kidney, are easily cultured and readily transfected [39]. Therefore they are extensively used in biotechnology and gene therapy to generate viral particles. HEK293T cells require splitting every other day using warm medium. They should not reach 100% confluency for optimal results. To have good transfection efficiency, these cells need to be cultured for at least one week to have them in log phase. Transfection efficiency was 22%, as determined based on the expression of tdTomato by fluorescence microscopy 24 hours later (Figure 7A-B). To generate a murine leukemia cell line expressing the tdTomato gene for immunotherapy studies, C1498 leukemic cells were transduced with freshly harvested virus (36 hours of transfection). Imaging studies (Figure 7C) and flow cytometric analysis (Figure 7D) four days after transduction confirmed the expression of tdTomato in the majority of the cells.

## Conclusions

In this manuscript, we describe a simple and step-by-step protocol explaining how to exploit the power of PCR to clone a GOI into a vector for genetic engineering. Several PCR-based creative methods have been developed being extremely helpful for the generation of new nucleotide sequences. This includes equimolar expression of several proteins by linking their genes via a self-cleaving 2A sequence [40, 41], engineering fusion proteins, as well as the use of linkers for the design of chimeric proteins [42–44]. Furthermore, protein tags [45, 46] and mutagenesis (site-directed, deletions, insertions) [47] have widened the applications of biological engineering. The protocol explained in this manuscript covers for most situations of PCR-assisted cloning; however, alternative PCR-based methods are available being restriction enzyme and ligation independent [6, 48–51]. They are of special interest in applications where restriction enzyme sites are lacking; nevertheless, these methods might need several rounds of PCR or occasionally a whole plasmid needs to be amplified. In such cases, the chance of PCR errors increases and necessitates sequencing of multiple clones. In conclusion, this guideline assembles a simple and straightforward protocol using resources that are tedious to collect on an individual basis thereby trying to minimize errors and pitfalls from the beginning.

## Methods

### Cell lines and media

The *E. coli* HB101 was used for the preparation of plasmid DNA. The bacteria were cultured in Luria-Bertani (LB) media. Human embryonic kidney (HEK) 293 T cells were cultured in Dulbecco’s Modified Eagle medium (DMEM) supplemented with 10% heat-inactivated fetal bovine serum (FBS), 1 mM sodium pyruvate, 0.1 mM nonessential amino acids, 2 mM L-glutamine, 100 mg/ml streptomycin, and 100 units/ml penicillin. A myeloid leukemia cell line C1498 [52], was cultured in Roswell Park Memorial Institute (RPMI) 1640 medium supplemented with the same reagents used for DMEM. Cells were split every other day to keep them on log phase.

### Plasmids, primers, PCR and sequencing

A plasmid containing the coding sequence of the tdTomato gene, plasmid containing an alpha-retroviral vector, and plasmids containing codon-optimized alpharetroviral gag/pol [53] were kindly provided by Axel Schambach (MHH Hannover, Germany). A forward (5′- ACCGGTGCCACCATGGCCACAACCATGGTG-3′) and a reverse (5′-GTCGACTTACTTGTACAGCTCGTCCATGCC-3′) primer used for the amplification of the tdTomato gene were synthesized by Eurofins Genomics (Ebersberg, Germany).

The optimal buffers for enzymes or other reagents were provided by the manufacturers along with the corresponding enzymes or inside the kits. If available by the manufacturers, the pH and ingredients of buffers are mentioned. Primers were dissolved in ultrapure water at a stock concentration of 20 pmol/μl. The template plasmid was diluted in water at a stock concentration of 50 ng/μl. For PCR, the following reagents were mixed and filled up with water to a total volume of 50 μl: 1 μl plasmid DNA (1 ng/μl final concentration), 1.25 μl of each primer (0.5 pmol/μl final concentration for each primer), 1 μL dNTP (10 mM each), 10 μl of 5X Phusion HF buffer (1X buffer provides 1.5 mM MgCl2), and 0.5 μl Phusion DNA polymerase (2U/μl, Thermo Scientific).

PCR was performed using a peqSTAR thermocycler (PEQLAB Biotechnologie) at: 98°C for 3 minutes; 25 cycles at 98°C for 10 seconds, 66°C for 30 seconds, 72°C for 30 seconds; and 72°C for 10 minutes. To prepare a 0.8% agarose gel, 0.96 g agarose (CARL ROTH) was dissolved in 120 ml 1X TAE buffer (40 mM Tris, 20 mM acetic acid, 1 mM EDTA, pH of 50X TAE: 8.4) and boiled for 4 minutes. Then 3 μl SafeView nucleic acid stain (NBS Biologicals) was added to the solution and the mixture was poured into a gel-casting tray.

DNA was mixed with 10 μl loading dye (6X) (Thermo Scientific) and loaded on the agarose gel (CARL ROTH) using 80 V for one hour in TAE buffer. The separated DNA fragments were visualized using an UV transilluminator (365 nm) and quickly cut to minimize the UV exposure. DNA was extracted from the gel slice using Zymoclean™ Gel DNA Recovery Kit (Zymo Research). The concentration of DNA was determined using a NanoDrop 2000 spectrophotometer (Thermo Scientific).

For sequence validation, the PCR product was subcloned using CloneJET PCR cloning kit (Thermo Scientific). 1 μl of blunt vector (50 ng/μl), 50 ng/μl of the PCR product, and 10 μl of 2X reaction buffer (provided in the kit) were mixed and filled with water to a total volume of 20 μl. 1 μl of T4 DNA ligase (5 U/μl) was added to the mixture, mixed and incubated at room temperature for 30 minutes. For bacterial transfection, 10 μl of the mixture was mixed with 100 μl of HB101 *E. coli* competent cells and incubated on ice for 45 minutes. Then the mixture was heat-shocked (42°C/2 minutes), put on ice again (5 minutes), filled up with 1 ml LB medium and incubated in a thermomixer (Eppendorf) for 45 minutes/37°C/450RPM. Then the bacteria were spun down for 4 minutes. The pellet was cultured overnight at 37°C on an agarose Petri dish containing 100 μg/mL of Ampicillin. The day after, colonies were picked and cultured overnight in 3 ml LB containing 100 μg/mL of ampicillin.

After 16 hours (overnight), the plasmid was isolated from the cultured bacteria using the QIAprep spin miniprep kit (QIAGEN) according to the manufacturer’s instructions. 720 to 1200 ng of plasmid DNA in a total of 12 μl water were sent for sequencing (Seqlab) in Eppendorf tubes. The sequencing primers pJET1.2-forward (5′-CGACTCACTATAGGGAG-3′), and pJET1.2-reverse (5′-ATCGATTTTCCATGGCAG-3′), were generated by the Seqlab Company (Göttingen, Germany). An ABI 3730XL DNA analyzer was used by the Seqlab Company to sequence the plasmids applying the Sanger method. Sequence results were analyzed using NCBI Blast as explained in the Results and discussion section.

### Manipulation of DNA fragments

For viewing plasmid maps, Clone Manager suite 6 software (SciEd) was used. Restriction endonuclease enzymes (Thermo Scientific) were used to cut plasmid DNA. 5 μg plasmid DNA, 2 μl buffer O (50 mM Tris–HCl (pH 7.5 at 37°C), 10 mM MgCl2, 100 mM NaCl, 0.1 mg/mL BSA, Thermo Scientific), 1 μl *Sal*I (10 U), and 1 μl *AgeI* (10 U) were mixed in a total of 20 μl water and incubated (37°C) overnight in an incubator to prevent evaporation and condensation of water under the tube lid. The next day, DNA was mixed with 4 μl loading dye (6X) (Thermo Scientific) and run on a 0.8% agarose gel at 80 V for one hour in TAE buffer. The agarose gel (120 ml) contained 3 μl SafeView nucleic acid stain (NBS Biologicals). The bands were visualized on a UV transilluminator (PEQLAB), using a wavelength of 365 nm, and quickly cut to minimize the UV damage. DNA was extracted from the gel slices using the Zymoclean™ gel DNA recovery kit (Zymo Research). The concentration of DNA was determined using a NanoDrop 2000 spectrophotometer (Thermo Scientific).

For the ligation of vector and insert fragments, a ligation calculator was designed (the Excel file available in the Additional file 1) for easy calculation of the required insert and vector volumes. The mathematical basis of the calculator is inserted into the excel spreadsheet. The size and concentration of the vector and insert fragments and the molar ratio of vector/insert (normally 1:3) must be provided for the calculation. Calculated amounts of insert (tdTomato) and vector (alpha-retroviral backbone) were mixed with 2 μl of 10X T4 ligase buffer (400 mM Tris–HCl, 100 mM MgCl2, 100 mM DTT, 5 mM ATP (pH 7.8 at 25°C), Thermo Scientific), 1 μl of T4 ligase (5 U/μl, Thermo Scientific), filled up to 20 μl using ultrapure water and incubated overnight at 16°C. The day after, HB101 *E. coli* was transfected with the ligation mixture as mentioned above. The clones were picked and consecutively cultured for one day in LB medium containing ampicillin. Plasmid DNA was isolated using Zyppy™ plasmid miniprep kit (Zymo Research) and digested with proper restriction enzymes for screening. Digested plasmids were mixed with the loading dye and run on an agarose gel as mentioned above. The separated DNA fragments were visualized using a Gel Doc™ XR+ System (BIO-RAD) and analyzed by the Image Lab™ software (BIO-RAD). The positive clone was cultured overnight in 450 ml LB medium containing ampicillin. Plasmid DNA was isolated using QIAGEN plasmid maxi kit (QIAGEN), diluted in ultrapure water and stored at −20°C for later use.

### Production of viral supernatant and transduction of cells

HEK293T cells were thawed, split every other day for one week and grown in log phase. The day before transfection, 3.5 × 10^6^ cells were seeded into tissue culture dishes (60.1 cm^2^ growth surface, TPP). The day after, the cells use to reach about 80% confluence. If over confluent, transfection efficiency decreases. The following plasmids were mixed in a total volume of 450 μl ultrapure water: codon-optimized alpharetroviral gag/pol (2.5 μg), VSVG envelope (1.5 μg), and the alpharetroviral vector containing the tdTomato gene (5 μg). Transfection was performed using calcium phosphate transfection kit (Sigma-Aldrich). 50 μl of 2.5 M CaCl_2_ was added to the plasmid DNA and the mixture was briefly vortexed. Then, 0.5 ml of 2X HEPES buffered saline (provided in the kit) was added to a 15 ml conical tube and the calcium-DNA mixture was added dropwise via air bubbling and incubated for 20 minutes at room temperature. The medium of the HEK293T cells was first replaced with 8 ml fresh medium (DMEM containing FCS and supplement as mentioned above) containing 25 μM chloroquine. Consecutively the transfection mixture was added. Plates were gently swirled and incubated at 37°C. After 12 hours, the medium was replaced with 6 ml of fresh RPMI containing 10% FCS and supplements. Virus was harvested 36 hours after transfection, passed through a Millex-GP filter with 0.22 μm pore size (Millipore), and used freshly to transduce C1498 cells. Before transduction, 24 well plates were coated with retronectin (Takara, 280 μl/well) for 2 hours at room temperature. Then, retronectin was removed and frozen for later use (it can be re-used at least five times) and 300 μl of PBS containing 2.5% bovine serum albumin (BSA) was added to the wells for 30 minutes at room temperature. To transduce C1498 cells, 5 × 10^4^ of cells were spun down and resuspended with 1 ml of fresh virus supernatant containing 4 μg/ml protamine sulfate. The BSA solution was removed from the prepared plates and plates were washed two times with 0.5 ml PBS. Then cells were added to the wells. Plates were centrifuged at 2000RPM/32°C/90 minutes. Fresh medium was added to the cells the day after.

### Flow cytometry and fluorescence microscope

For flow cytometry assessment, cells were resuspended in PBS containing 0.5% BSA and 2 mM EDTA and were acquired by a BD FACSCanto™ (BD Biosciences) flow cytometer. Flow cytometry data were analyzed using FlowJo software (Tree Star). Imaging was performed with an Olympus IX71 fluorescent microscope equipped with a DP71 camera (Olympus). Images were analyzed with AxioVision software (Zeiss). Fluorescent images were superimposed on bright-field images using adobe Photoshop CS4 software (Adobe).

## Electronic supplementary material

Additional file 1: **Ligation calculator.** To calculate the amounts of the vector and insert fragments for a ligation reaction, you need to provide the size of the vector and insert (in base pairs), the molar ration of insert/vector (normally 3 to 5), vector amount (normally 50 to 100 ng), and vector and insert fragment concentrations (ng/μl). The computational basis of this ligation calculator is mentioned in the lower box. (XLSX 50 KB)

## Abbreviations

PCR: Polymerase chain reaction
GOI: Gene of interest
ORF: Open reading frame
Tm: Melting temperature
BLAST: Basic local alignment search tool
VSVG: Vesicular stomatitis virus G glycoprotein
LB: Luria-Bertani
DMEM: Dulbecco’s Modified Eagle medium
RPMI: Roswell Park Memorial Institute
BSA: Bovine serum albumin
EDTA: Ethylenediaminetetraacetic acid
FACS: Fluorescence-activated cell sorting
HEK: Human embryonic kidney
PBS: Phosphate buffered saline
FCS: Fetal calf serum
HEPES: Hydroxyethyl-piperazineethane-sulfonic acid
AMP: Ampicillin resistance gene
PRE: Posttranscriptional regulatory element
MPSV: Myeloproliferative sarcoma virus promoter.
